# The impact of positive-pressure breathing apparatus on muscle fatigue of volunteer firefighter

**DOI:** 10.1371/journal.pone.0305599

**Published:** 2024-06-24

**Authors:** Huimin Hu, Jie Wang, Lixin Ouyang, Ling Luo, Wenlei Niu

**Affiliations:** 1 Ergonomics Laboratory, China National Institute of Standardization, Beijing, China; 2 SAMR Key Laboratory of Human Factors and Ergonomics, Beijing, China; 3 School of Management Engineering, Capital University of Economics and Business, Beijing, China; 4 CASCO SIGNAL LTD, Beijing, China; University of Mississippi, UNITED STATES

## Abstract

Muscle fatigue is one of the leading causes that contributes tremendously to injuries among volunteer firefighters in the workplace. The purpose of this study was to investigate the impact of positive-pressure breathing apparatus on muscle fatigue in the shoulder, back, and legs of volunteer firefighters. A total of 60 volunteer firefighters were recruited to perform a running task on a motorized treadmill in a controlled laboratory environment. Surface electromyography and rating of perceived exertion scores were collected from all participants every 60 seconds during the running task. Results show that the median frequency values for all measured muscle groups were significantly lower, and the rating of perceived exertion score was significantly higher after running with the positive-pressure breathing apparatus. Meanwhile, there were no significant differences in the median frequency values for the upper trapezius, erector spinae, and biceps femoris between the initial and final periods of running task without load. However, the median frequency values with load for gastrocnemius, rectus femoris, and tibialis anterior exhibited a greater downward trend compared to those without load. Additionally, using a breathing apparatus can cause asymmetric muscle fatigue in bilateral upper trapezius, erector spinae, gastrocnemius, and tibialis anterior muscles. The decreased performance due to muscle fatigue increases the risk of accidents, thereby posing a threat to the safety of volunteer firefighters. This study offers valuable insights into the effects of positive-pressure breathing apparatus on muscle fatigue among volunteer firefighters. These results may serve as a reference for developing improved fatigue management strategies and optimizing the design features of breathing apparatus.

## Introduction

The “14th Five-Year Plan” national fire protection work plan in China points out that the current fire safety situation in China is still grim and proposes higher standards and stricter requirements for various types of firefighters [1]. Volunteer firefighters, as an important part of the firefighter team, are generally concurrently served by staff from the safety management department and work for the miniature fire station in institutions, enterprises or townships [2]. This means that volunteer firefighters do not receive as much strength and fitness training as professional firefighters. According to the requirements of the Beijing’s local standard “Code for the Construction of Small Fire Stations” (DB11/T 1483–2017), volunteer firefighters commonly carry positive-pressure breathing apparatus when fighting a fire and wear 10 other items of personal protective equipment, such as fire-retardant boots, jackets, gloves, helmets [3]. The additional weight of these equipment can be up to 35 pounds. Miniature fire stations are usually equipped with several 6.8L positive-pressure breathing apparatuses, each weighing approximately 22 pounds. While these apparatuses can serve to safeguard the respiratory system of volunteer firefighters from toxic and harmful substances, they also impose a significant physical burden on their shoulders and lower back, resulting in muscle fatigue.

Muscle fatigue often manifests as a decrease in muscle contraction rate [4], a decrease in force output [5], and a subjective perception of increase difficulty exerting certain forces or completing specific action [6]. Fatigue-induced performance degradation heightens the risk of accidents, thereby jeopardizing personal health and safety [7]. Muscle fatigue can lead to occupational diseases [8] such as musculoskeletal disorders [9], cardiovascular disease [10], and mental health disorders [11]. A statistical study identified fatigue as a significant contributor to workplace injuries with overexertion accounting for 33% of all firefighter injuries [12]. The incidence of low back and shoulder injuries among firefighters is also notably higher than that of the general population [13]. The U.S. National Fire Protection Association (NFPA) conducted research on fireground injuries experienced by US firefighters and found that from 2017 to 2021, overexertion or strain accounted for 21% of the injuries [14], while in 2022, strain, sprain, and muscular pain accounted for 56% of all reported injuries [15]. Furthermore, volunteer firefighters experienced overexertion or strain as the most common type of injury, comprising 26% of all reported cases [16]. Ras et al. conducted a systematic review and meta-analysis to assess the effects on occupational performance among firefighters [17] and discovered a significant impact of physical fitness on occupational performance through muscular endurance [18] and upper body strength [19]. Statistical reports and existing studies have consistently shown that fatigue plays a significant role in fireground injuries. Alongside the dangerous and complex environment, they work in, volunteer firefighters are burdened with heavy personal protective equipment such as positive-pressure breathing apparatus, which significantly contribute to muscle fatigue [20]. Bakri et al. investigated the impact of breathing apparatus weight on physiological responses and subjective experiences, concluding that increasing weight enhance subjective muscle fatigue and discomfort [21].

Previous research has established that excessive load placed on the firefighters can result in physiological burdens [22–25]. In addition, studies have been conducted to examine the impact of personal protective clothing and breathing apparatus on various physiological indicators (e.g., maximal oxygen uptake, heart rate, blood lactate concentration, peak exercise) in firefighters [26–28]. In comparison to professional firefighters, volunteer firefighters in China often do not undergo standardized fitness and strength training. However, little attention has been paid to the muscle fatigue in volunteer firefighters caused by the load of a breathing apparatus.

In general, surface electromyography (sEMG) is an important measure for assessing muscle fatigue [29]. The median frequency (MF), derived from the sEMG signal, is one of the most frequently used parameters [30]. Prior research has suggested that a decrease in MF values indicates the onset of muscle fatigue [31]. When muscle fatigue occurs, it is often reflected in both physical fatigue and subjective fatigue perception [32]. Therefore, it is necessary to examine the subjective perception of fatigue among volunteer firefighters when they are carrying heavy loads.

The aim of this study was to examine muscle fatigue in the shoulder, back, and legs of volunteer firefighters while performing running tasks with a positive-pressure breathing apparatus. This motion is frequently employed in their work. Based on literature reviews and field investigations, major muscle groups including upper trapezius, erector spinae, biceps femoris, gastrocnemius, rectus femoris and tibialis anterior were selected for analysis. These muscles are closely associated with overuse injuries, strain, and sprain [14, 15].

Using sEMG measurements, the differences in fatigues between load conditions (with the breathing apparatus) and non-load conditions (without the breathing apparatus) were analyzed for these selected muscle groups. Additionally, the rating of perceived exertion (RPE) scale was employed here to assess subjective feelings of exhaustion among volunteer firefighters. The experimental results serve to ascertain whether the physical capabilities of volunteer firefighters are sufficient and provide reference points and suggestions for fitness and strength training plans. They also provide a basis for improving fatigue and optimizing the design of air breathing apparatus.

## Materials and methods

### Subjects

Sixty healthy male volunteer firefighters participated in the study for one non-load trial and one loaded trial. All of them were from the miniature fire station of the Changping Experimental Base of the China National Institute of Standardization and other nearby institutions, meeting the occupational health standards for volunteer firefighters [33]. The average age was 22.6 ± 1.82 years, height was 176.2 ± 7.22 cm, and body weight was 72.8 ± 10.27 kg (mean ± SD). None of the subjects had any injuries, severe medical conditions or surgeries in the past six months. After fully comprehending the aim of the study and signing informed consent, they chose to take part in the experiment. All subjects received training to get familiar with the experimental procedures and were capable of completing the experiment on their own. Only essential information and data pertinent to the study’s purpose were gathered and stored anonymously to safeguard the privacy of the participants. The experimental protocol was approved by the Institutional Review Board of the China National Institute of Standardization.

### Experimental protocols and procedures

All subjects were required to visit the laboratory twice to complete the experiment, which involved non-load and load conditions. The interval between two trials was 48 hours. Before each trial, subjects were advised to refrain from alcohol and intense physical activity, as well as to avoid consuming food, drugs, and caffeine for 2 hours.

**Fig 1:**
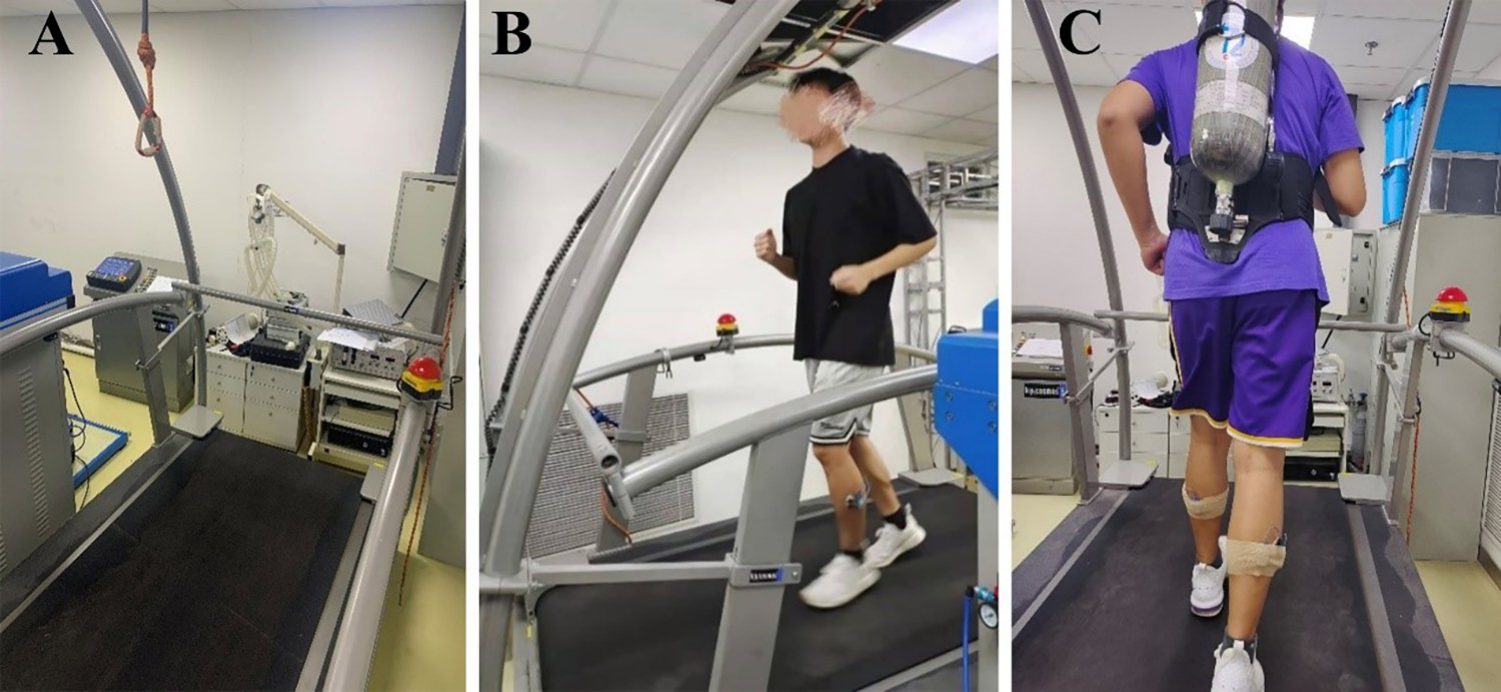
Experimental conditions. A: treadmill and safety rope; B: test without breathing apparatus (non-load); C: test with breathing apparatus (load).

The experiment was conducted on a motorized treadmill (h/p/cosmos mercury; Fiszman Medical Electronics GMBH; Germany) in the ergonomics laboratory of the Changping Experimental Base. During the trial, the room temperature was maintained at 26°C with natural ventilation. For the load condition test, a commonly used 6.8L air breathing apparatus was utilized. Additionally, a suspended safety rope was installed to ensure the safety of participants during high-speed running (Fig 1).

It was necessary to have a full rest and sufficient warm-up before the trial. When subjects visited the laboratory, they were instructed to rest for 15 minutes on a chair and then warm up with a 5-minute walk at a low speed of 4 km/hr (without incline and non-load).

After the warm-up period, subjects were instructed to rate their perceived exertion using the RPE Scale (Rating of perceived exertion) before commencing the test. The RPE Scale is a scale that ranges from 1, indicating little to no effort, to 10, indicating maximum effort [34]. The running test was started without inclination. The treadmill speed of the test was increased gradually:

(1) During the initial speed phase, the treadmill was set to a speed of 4 km/hr, and subjects were required to walk at this speed for 60 seconds.

(2) In the acceleration phase, the speed was incremented by 0.1 km/hr every two seconds up to 8 km/hr. This was then followed by further increases of 0.1 km/hr every second until reaching 10 km/hr. The phase spanned a total duration of 100 seconds.

(3) Throughout the constant speed phase, subjects were encouraged to maintain a speed of 10 km/hr for a duration of 15 minutes. However, if subjects experienced intolerable muscle fatigue and could not continue, the test was terminated prematurely.

Following the running task, subjects continued to walk on the treadmill at a comfortable speed until they decided to stop in order to alleviate the physiological discomfort associated with an abrupt cessation.

To minimize the carryover effect between the two trials (non-load and loaded), the sequence of conditions was counterbalanced among the subjects. Thirty volunteer firefighters began by performing the running task without the positive-pressure breathing apparatus, while others started with the load trial, carrying the heavy positive-pressure breathing apparatus. A 48-hour rest period was provided between trials.

### Measurements

**Fig 2:**
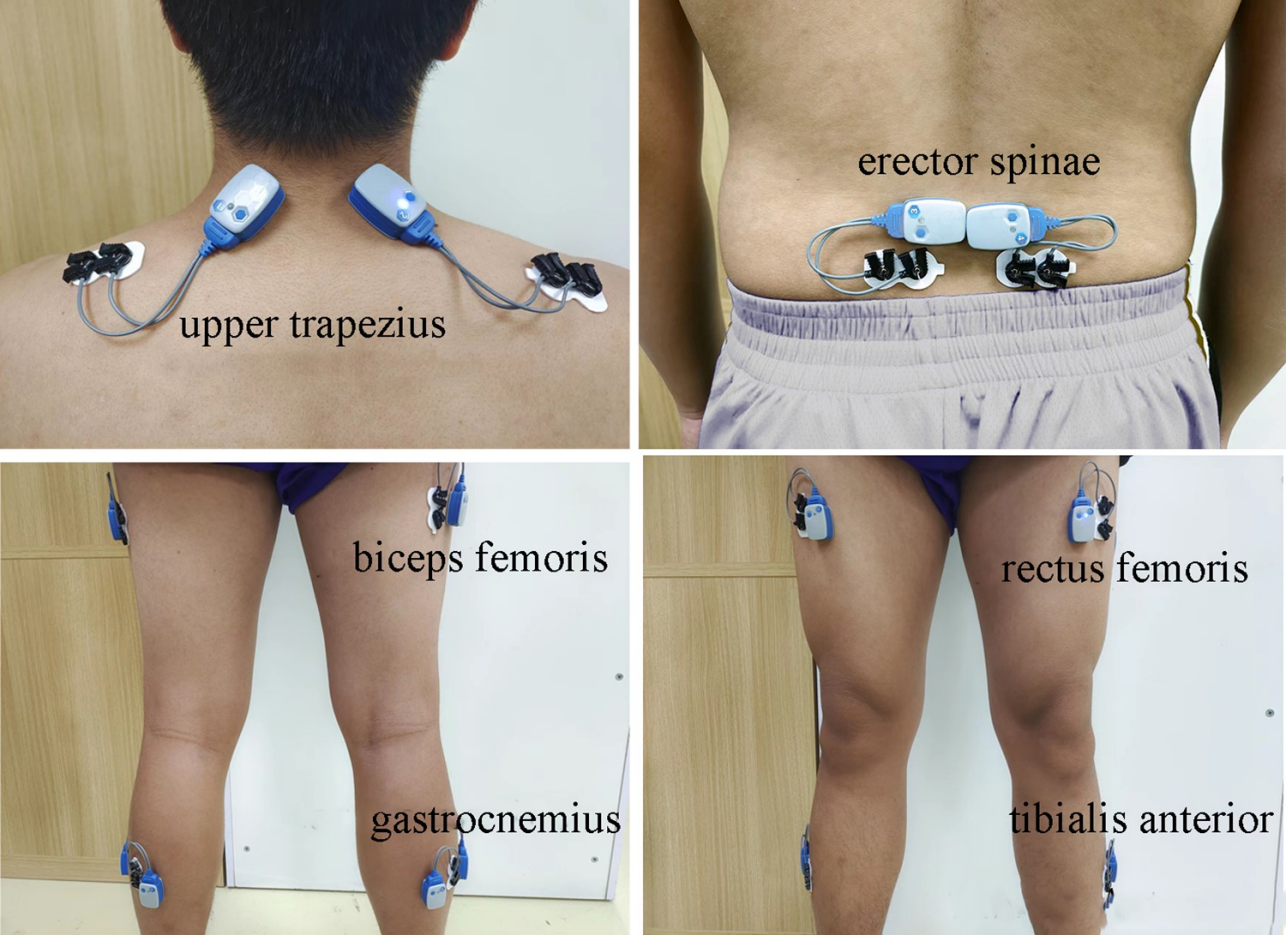
sEMG measuring positions.

A 16-channel wireless sEMG signal acquisition and analysis system (Ultium ESP; Noraxon; U.S.) was used to record the sEMG signals from the shoulder, back and legs at a sampling frequency of 4000 Hz. As shown in Fig 2, the sEMG sensors were placed on bilateral upper trapezius, erector spinae, biceps femoris, gastrocnemius, rectus femoris and tibialis anterior according to SENIAM guidelines [35]. The center-to-center distance between the two recording electrodes was 2 cm. Prior to this, the relevant skin areas were disinfected, shaved and cleaned to minimize unnecessary noise and error.

The sEMG data were collected at the beginning of both the initial speed phase and the constant speed phase. Subsequently, the sEMG data were collected every 60 seconds until the end of the running task with each collection lasting 20 seconds. In addition, the RPE scores of subjects were collected every 60 seconds during the constant speed phase.

### Data presentation and statistical analysis

In this experiment, 720 sets of sEMG data (60 subjects × 6 muscle groups × 2 loads) were recorded. The raw sEMG data were analyzed using the myoMUSCLE™ Software module (MR 3.16.102, Noraxon; U.S.). the MF eigenvalues were selected for muscle fatigue evaluation in our study based on previous research [36]. Data pre-processing and the calculation of MF eigenvalues were conducted using MATLAB 2018 (MathWorks; U.S.). A second-order notch filter with a 49–51 Hz band stop was used to eliminate AC line interference. The wavelet function db4 and the 6th decomposition layer were chosen to separate noise from the original signals [37]. After noise reduction, rectification, filtering, smoothing and standardization, the MF in frequency domain feature analysis was extracted as the fatigue feature parameter using the following Eq (1).

$$\int_{0}^{MF}S(f)df=\int_{MF}^{\infty }S(f)df=\frac{1}{2}\int_{0}^{\infty }S(f)df$$ (1)

Where MF represents the median frequency values, *f* denotes the frequency, *S*(*f*) signifies the power spectral density function, and *df* is the frequency resolution [38].

The MF values collected for each period were presented in the form of mean and standard deviation (SD). Although subjects were instructed to perform a 15-minute running task at a speed of 10 km/hr, in actuality, only 13 participants were able to run for more than seven minutes with the positive-pressure breathing apparatus. Therefore, the MF of the first seven times (at the time of 0 min, 1 min, 2 min, 3 min, 4 min, 5 min and 6 min) of the constant speed phase were selected for fatigue analysis.

For statistical analysis, the Wilcoxon signed-rank test was conducted to determine whether the MF values at the initial period (0 min) and the end of the test (6 min) during the constant speed phase were significantly different. The difference in MF values between load and non-load conditions was also identified using the Wilcoxon signed-rank test. Additionally, the Mann-Whitney U test was performed to determine whether there was a significant difference in the degree of bilateral muscle fatigue. A decrease in MF can indicate the onset of muscle fatigue [31, 39]. The mean MF of the first 6 minutes during the constant speed phase was depicted as a dot plot for linear regression analysis to observe changes in muscle group fatigue.

The RPE scores of subjects, collected at each period, were also presented in the form of mean and standard deviation (SD). The difference in RPE values between non-load and load conditions was analyzed using a paired t-test. The subjects’ fatigue experienced under non-load and load conditions was analyzed by comparing the difference in RPE values.

The RPE values from the initial 6 minutes of the constant speed phase were analyzed to compare differences in subjective responses to fatigue. All statistical analyses were performed using SPSS 2019 (version 19.0; IBM SPSS Statistics, Somers, N.Y.) with a confidence interval of 95%.

## Result

**Fig 3:**
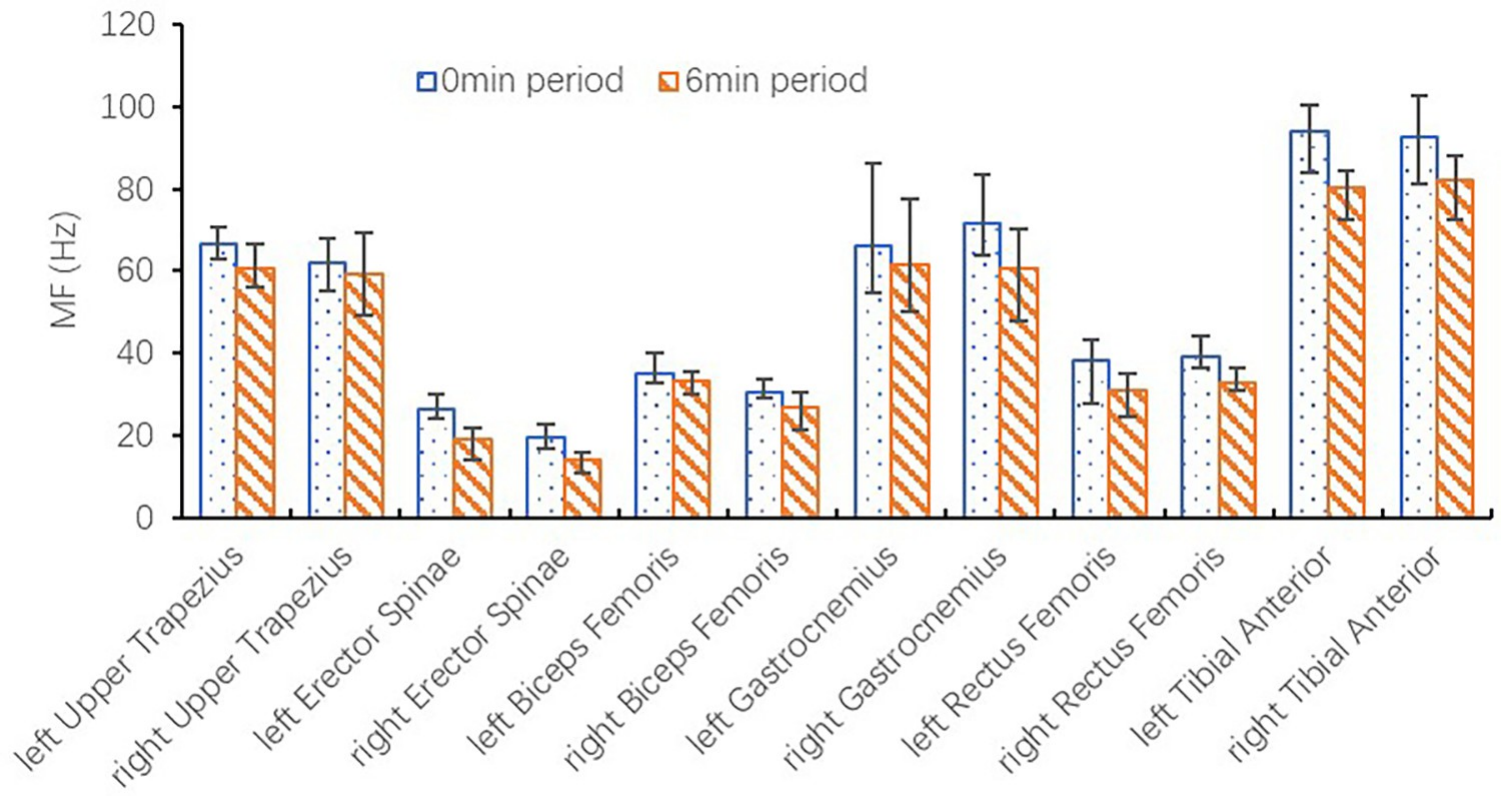
Bilateral MF values of the subject during the constant speed phase (with load).

**Fig 4:**
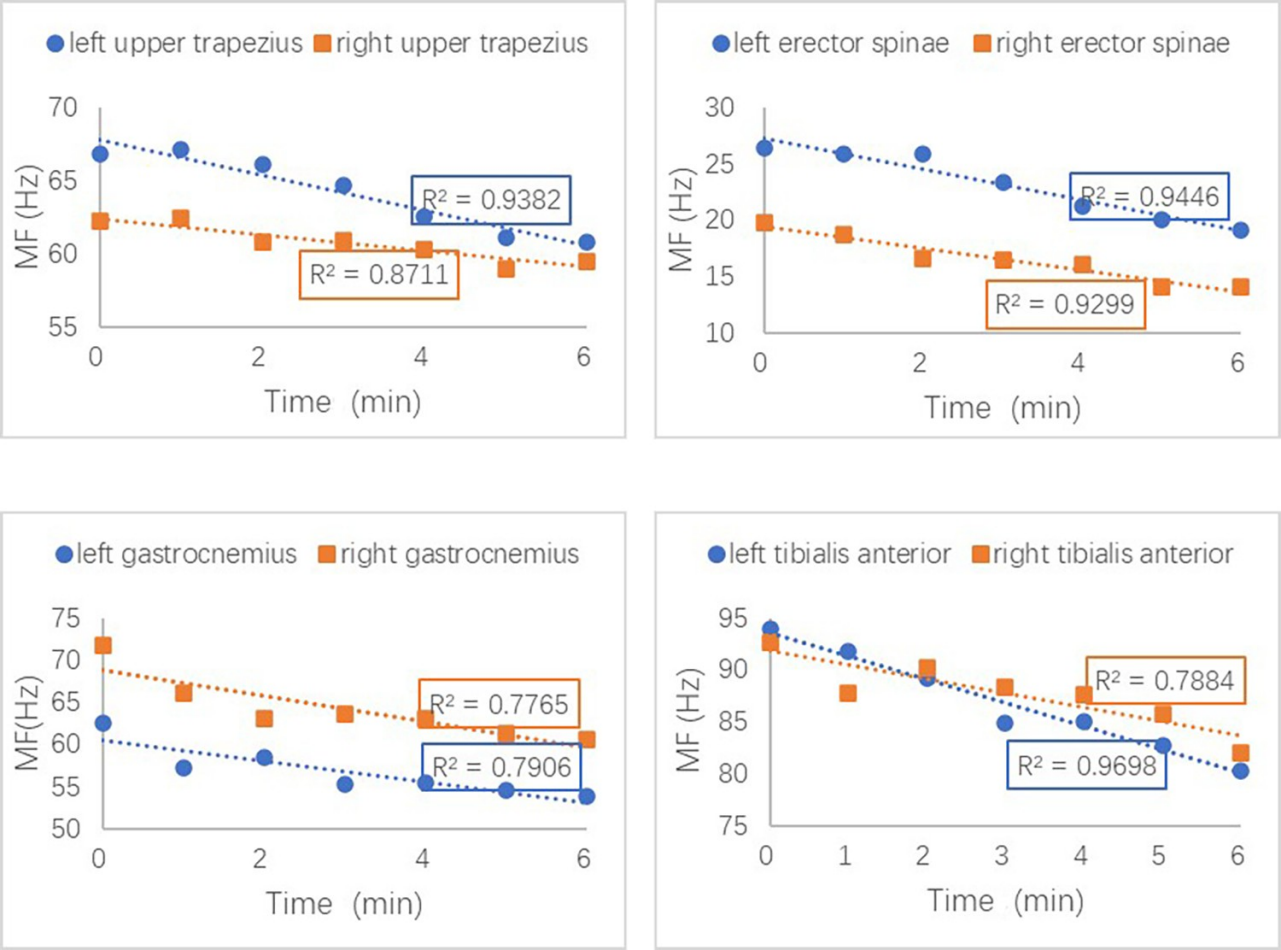
Changes in MF values of four muscle groups between the left and the right during the constant speed phase under load conditions.

As shown in Fig 3, the MF eigenvalues of all muscle groups measured at the 6-minute period were significantly lower than those recorded at the 0-minute period during the constant speed phase of running with a load (p<0.001). For instance, there was a significant decrease in the MF of the right gastrocnemius, left tibialis anterior, and right tibialis anterior by 11.13 ± 5.28 Hz, 13.70 ± 4.23 Hz, and 10.52 ± 5.75 Hz respectively, indicating fatigue. Although no significant differences were observed in the MF eigenvalues of the bilateral upper trapezius, erector spinae, and biceps femoris (p = 0.594) without a load. The difference in MF values for the bilateral upper trapezius, erector spinae, biceps femoris, gastrocnemius, rectus femoris, and tibialis anterior during the constant speed phase was analyzed under both non-load and load conditions. It was found that the MF values of the left upper trapezius, left erector spinae, right gastrocnemius, and left tibialis anterior under load conditions decreased more than those on the contralateral side (p<0.001). Meanwhile, the reduction in MF values of all muscle groups under non-load conditions between the left and right sides did not show a significant difference (p = 0.375). As shown in Fig 4, the changes in upper trapezius, erector spinae, gastrocnemius, and tibialis anterior are represented by a linear regression fit analysis, which demonstrates a good goodness of fit (R^2^ > 0.6), and the downward trend difference of bilateral MF values is significant.

**Fig 5:**
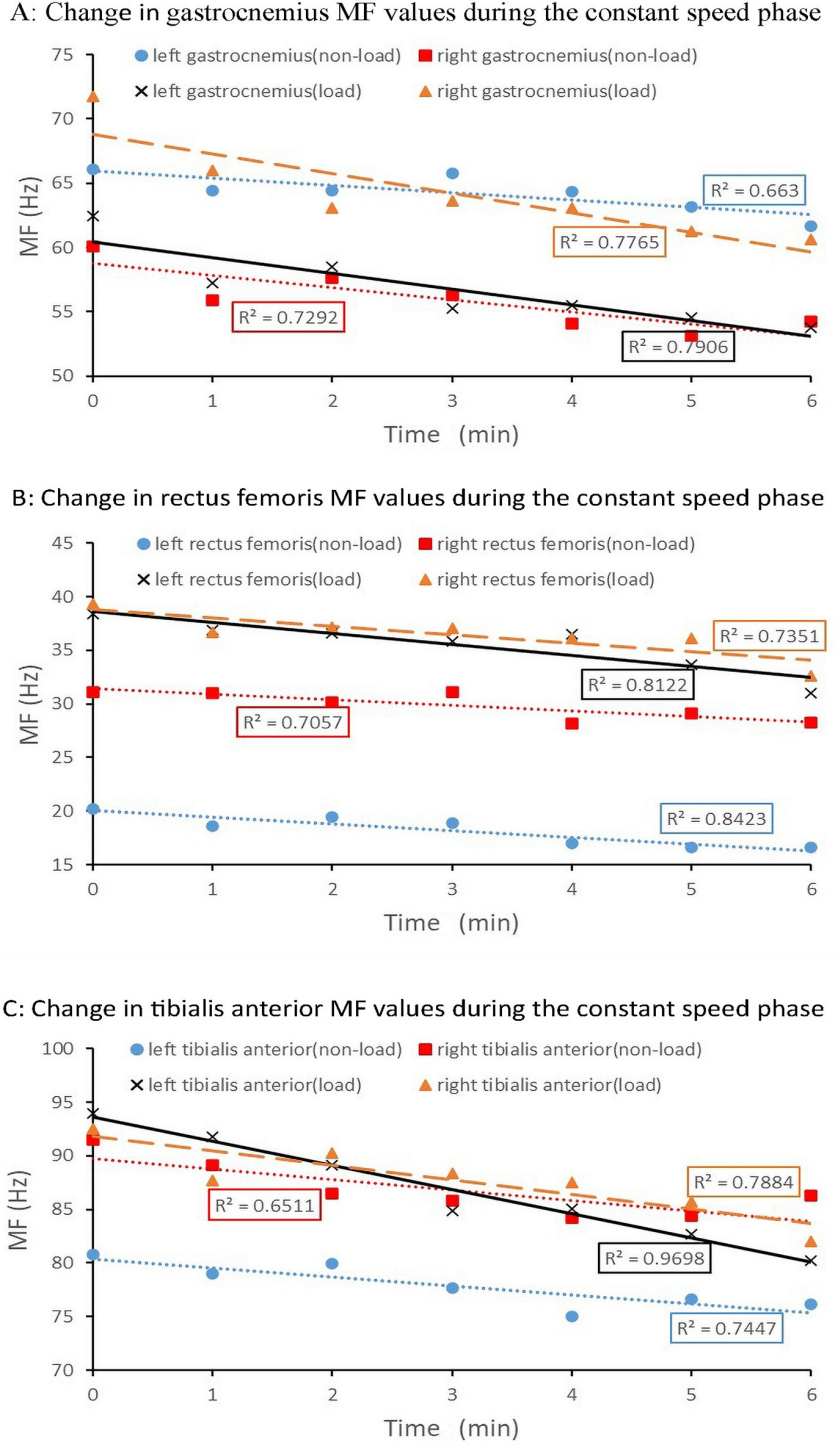
Changes in MF values of three muscle groups in the legs during the constant speed phase under non-load and load conditions.

The eigenvalues of MF for three leg muscle groups—bilateral gastrocnemius, rectus femoris and tibialis anterior—decreased significantly without load (p = 0.010). However, these changes were less pronounced compared to when carrying the positive-pressure breathing apparatus. As shown in Fig 5, a linear regression fit analysis with a high goodness of fit (R^2^ > 0.6) reveals a significant downward trend in MF values under both load and non-load conditions. Nevertheless, this downward trend is more pronounced under the load condition.

**Fig 6:**
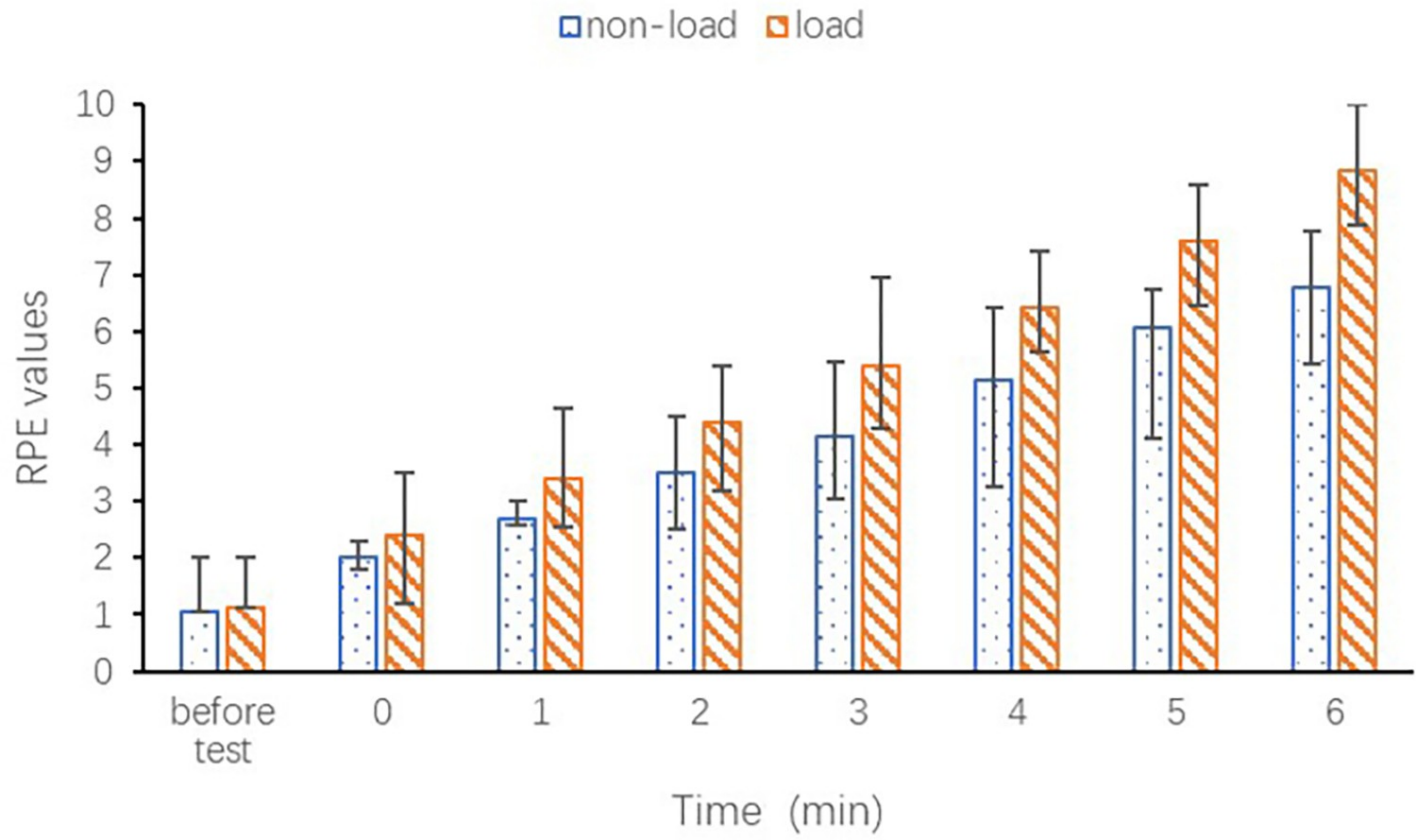
Changes in Rating of Perceived Exertion (RPE) under non-load and load conditions.

The study compared fatigue levels experienced before and after the test, as well as without and with the use of a positive-pressure breathing apparatus, as depicted in Fig 6. The difference in fatigue between the two experimental conditions was not significant (p = 0.700) before the experiment. After the running task, the RPE values significantly increased under both non-load and load conditions (p<0.001). At the end of the running task (6 min), the RPE values were significantly higher under the load condition compared to the non-load condition (6.79 ± 1.16 vs. 8.84 ± 1.07 for non-load and load, respectively; p<0.001). These findings suggest that running while wearing with a positive-pressure breathing apparatus resulted in greater subjective fatigue among volunteer firefighters, which is consistent with the sEMG results.

## Discussion

Muscle fatigue is more broadly defined as a decline in muscle endurance and muscle strength due to exercise. When muscles are fatigued, their force-generating capacity decreases [40]. The purpose of our study was to determine the fatigue levels of specific muscles—namely, the upper trapezius, erector spinae, biceps femoris, gastrocnemius, rectus femoris, and tibialis anterior—in volunteer firefighters while they carried out tasks wearing positive-pressure breathing apparatus, using sEMG analysis [41, 42]. The results showed that the degree of muscle fatigue significantly increased after the completion of the selected running tasks. It also demonstrated that the Bruce treadmill protocol [43] is a valuable method for identifying the level of aerobic endurance in volunteer firefighters and can be used for future fatigue assessments.

Moreover, the fatigue levels were significantly higher in the loaded running condition than those in the non-loaded condition for the upper trapezius, erector spinae, and biceps femoris. furthermore, the perceived fatigue, as indicated by the RPE values, was significantly higher in the loaded condition than those in the non-loaded condition. Our study’s findings are consistent with previous research that have shown that increasing load and exercise duration can exacerbate muscle fatigue [44]. Since our subjects were volunteer firefighters in China who often lack standardized fitness and strength training, it appears that their current training is inadequate to prevent a significant increase in both muscular and perceived fatigue during the loaded running task. The experimental results highlight the necessity for establishing a standardized physical training program for volunteer firefighters. Such a program should include exercises like weight-bearing running to ensure they can perform tasks without experiencing excessive fatigue while using various types of firefighting equipment (e.g. air breathing apparatus).

The test was intended for participants to run at a speed of 10 km/hr for 15 minutes. However, in practice, only a few participants were able to run for more than 7 minutes under the load of the breathing apparatus. Nonetheless, all subjects were able to complete the 15-minute running task without any additional load. Therefore, only data from the first 6 minutes of the constant speed phase were used for comparative analysis. Volunteer firefighters also require good physical fitness and strength when working on firegrounds with heavy personal protective equipment. This study showed that volunteer firefighters were unable to maintain a long-term work with the breathing apparatus due to muscle and perceived fatigue. Physical work capacity is an important factor affecting their performance, and muscle fatigue can reduce this capacity, posing risks to the individual firefighter, colleague or victim [45]. Dawson et al. suggest organizing a systematic and unified approach to managing fatigue among volunteer firefighters in the emergency services sector [46]. Health and fitness programs specifically tailored for volunteer firefighters should include firefighter-specific exercise programs as essential components. Additionally, it is necessary to adopt certain recovery modality that enhances muscle relaxation and quickly alleviates fatigue among volunteer firefighters. Ilham et al.’s research found that improving muscle fatigue among firefighters could be achieved through lightweight design of the breathing apparatus and optimization of harness designs [21]. Therefore, future studies on the effect of physical fitness training or the design of lightweight breathing apparatus on reducing muscle and perceived fatigue in volunteer firefighters are needed.

In our study, we also observed varying degrees of decrease in MF values among bilateral muscle groups. When running with a breathing apparatus, the left upper trapezius, left erector spinae, right gastrocnemius and left tibialis anterior exhibited greater fatigue compared to their contralateral muscles. Meanwhile, muscle fatigue under non-load condition did not show a significant difference between the left and right sides. Previous studies have indicated potential changes in the center of gravity of the firefighter equipment during walking, running and other body movements [47]. Consequently, with such a changing position of the gear, it is speculated that the observed asymmetry of the muscle fatigue may further affect the mobility, postural control and balance performance of volunteer firefighters when they are required to work with the positive-pressure breathing apparatus [48].

For convenience, all volunteer firefighters in this study were male, aged 22.6 ± 1.82 years, from the miniature fire station near the laboratory. However, it should be noted that there are actually many female volunteer firefighters and volunteers of different age range. Therefore, these results have limitations and further research is needed to include female and older volunteer firefighters. In order for the volunteer firefighters experienced muscle fatigue as quickly as possible without being injured during the experiment, a running speed of 10 km/hr was chosen based on the Bruce treadmill protocol [43]. However, it should be noted that volunteer firefighters’ work involves various movements such as sprinting, variable speed running, jumping, bending, lifting, and pushing. Therefore, future studies could introduce more task scenarios to investigate muscle fatigue caused by breathing apparatus. Additionally, differences in weight, training level, height, and other factors among volunteer firefighters may influence the development of muscle fatigue when using air breathing apparatus.

Since fatigue can arise from both the central and cardiovascular systems, as well as from muscular exhaustion, it is important to consider the high RPE values observed in this study were not solely due to muscle fatigue. To minimize confounding factors, participants were required to wear lightweight clothing during the experiment. However, it should be acknowledged that volunteer firefighters typically wear heavy protective clothing and boots during fire emergencies. Therefore, further study should also incorporate physiological indicators (e.g., maximal oxygen uptake, heart rate, blood lactate concentration, peak exercise) to better understand how personal protective equipment and breathing apparatus affect volunteer firefighters’ fatigue.

## Supporting information

S1 File Data. (XLSX)
